# Assessment of Patient-Centered Approaches to Collect Sexual Orientation and Gender Identity Information in the Emergency Department: The EQUALITY Study

**DOI:** 10.1001/jamanetworkopen.2018.6506

**Published:** 2018-12-28

**Authors:** Adil Haider, Rachel R. Adler, Eric Schneider, Tarsicio Uribe Leitz, Anju Ranjit, Christina Ta, Adele Levine, Omar Harfouch, Danielle Pelaez, Lisa Kodadek, Laura Vail, Claire Snyder, Danielle German, Susan Peterson, Jeremiah D. Schuur, Brandyn D. Lau

**Affiliations:** 1 Center for Surgery and Public Health, Brigham & Women’s Hospital, Harvard Medical School, Boston, Massachusetts; 2 Harvard T.H. Chan School of Public Health, Boston, Massachusetts; 3 School of Medicine and Public Health, University of Wisconsin, Madison; 4 University of Virginia, Charlottesville; 5 Johns Hopkins University School of Medicine, Baltimore, Maryland; 6 Johns Hopkins Bloomberg School of Public Health, Baltimore, Maryland; 7 Brigham & Women’s Hospital, Boston, Massachusetts; 8 Harvard Medical School, Boston, Massachusetts

## Abstract

**Importance:**

Health care and government organizations call for routine collection of sexual orientation and gender identity (SOGI) information in the clinical setting, yet patient preferences for collection methods remain unknown.

**Objective:**

To assess of the optimal patient-centered approach for SOGI collection in the emergency department (ED) setting.

**Design, Setting, and Participants:**

This matched cohort study (Emergency Department Query for Patient-Centered Approaches to Sexual Orientation and Gender Identity [EQUALITY] Study) of 4 EDs on the east coast of the United States sequentially tested 2 different SOGI collection approaches between February 2016 and March 2017. Multivariable ordered logistic regression was used to assess whether either SOGI collection method was associated with higher patient satisfaction with their ED experience. Eligible adults older than 18 years who identified as a sexual or gender minority (SGM) were enrolled and then matched 1 to 1 by age (aged ≥5 years) and illness severity (Emergency Severity Index score ±1) to patients who identified as heterosexual and cisgender (non-SGM), and to patients whose SOGI information was missing (blank field). Patients who identified as SGM, non-SGM, or had a blank field were invited to complete surveys about their ED visit. Data analysis was conducted from April 2017 to November 2017.

**Interventions:**

Two SOGI collection approaches were tested: nurse verbal collection during the clinical encounter vs nonverbal collection during patient registration. The ED physicians, physician assistants, nurses, and registrars received education and training on sexual or gender minority health disparities and terminology prior to and throughout the intervention period.

**Main Outcomes and Measures:**

A detailed survey, developed with input of a stakeholder advisory board, which included a modified Communication Climate Assessment Toolkit score and additional patient satisfaction measures.

**Results:**

A total of 540 enrolled patients were analyzed; the mean age was 36.4 years and 66.5% of those who identified their gender were female. Sexual or gender minority patients had significantly better Communication Climate Assessment Toolkit scores with nonverbal registrar form collection compared with nurse verbal collection (mean [SD], 95.6 [11.9] vs 89.5 [20.5]; *P* = .03). No significant differences between the 2 approaches were found among non-SGM patients (mean [SD], 91.8 [18.9] vs 93.2 [13.6]; *P* = .59) or those with a blank field (92.7 [15.9] vs 93.6 [14.7]; *P* = .70). After adjusting for age, race, illness severity, and site, SGM patients had 2.57 (95% CI, 1.13-5.82) increased odds of a better Communication Climate Assessment Toolkit score category during form collection compared with verbal collection.

**Conclusions and Relevance:**

Sexual or gender minority patients reported greater comfort and improved communication when SOGI was collected via nonverbal self-report. Registrar form collection was the optimal patient-centered method for collecting SOGI information in the ED.

## Key Points

**Question:**

What is the optimal patient-centered approach to collecting sexual orientation and gender identity information in the emergency department?

**Findings:**

In this matched cohort study of 540 adults, sexual and gender minority patients reported significantly higher patient satisfaction with registrar form collection compared with nurse verbal collection. Non–sexual and gender minority patients, in addition to those for whom sexual orientation and gender identity information was not collected, reported no worse outcomes with registrar form collection.

**Meaning:**

Registrar form collection is the optimal patient-centered approach to collecting sexual orientation and gender identity information in the emergency department.

## Teaser

This matched cohort study assesses the optimal patient-centered approach for sexual orientation and gender identity collection in the emergency department setting, comparing nurse verbal collection during the clinical encounter vs nonverbal collection during patient registration.

## Introduction

Four percent of the US population identifies as sexual and gender minorities (SGM), including identities such as lesbian, gay, bisexual, and/or transgender (LGBT).^1^ Recent estimates indicate that the number of American adults identifying as SGM has increased from 8 million in 2012 to 10 million in 2016.^1^ Physical^2,3^ and mental^4,5^ health inequities as well as health care access disparities^3,6^ among SGM exist; however, the magnitude of these disparities has not been fully determined owing to lack of routine collection of patients’ sexual orientation and gender identity (SOGI) data.

Given the need to further understand health disparities among SGM individuals, The Joint Commission^7^ and the Department of Health and Human Services^8^ recommend routine collection of SOGI information in health care settings. Meaningful Use Stage 3 Guidelines^9^ mandate that any health care facility that uses electronic health records (EHRs) has the ability to collect SOGIs. However, even with these directives for SOGI collection, little work has been done to understand patient preferences for SOGI collection, especially within the emergency department (ED) setting. We initiated the Emergency Department Query for Patient-Centered Approaches to Sexual Orientation and Gender Identity (EQUALITY) Study to identify the optimal patient-centered method of collecting SOGI information from patients in the ED.

Phase 1 of the EQUALITY Study revealed that approximately 80% of clinicians believe patients would refuse to provide SOGI, yet only 10% of patients reported they would refuse to do so.^10^ Standardized collection of SOGI is viewed by SGM patients as a step toward recognition as an individual as well as normalization of SGM individuals within society.^11^ Although early results indicated that patients are willing to provide SOGI information, the preferred method of collection in the ED setting remained unclear. We describe results from an interventional study (the EQUALITY Study) that compared 2 different patient-centered methods of collecting SOGI information in the ED.

## Methods

### Study Design and Oversight

**Figure 1.**
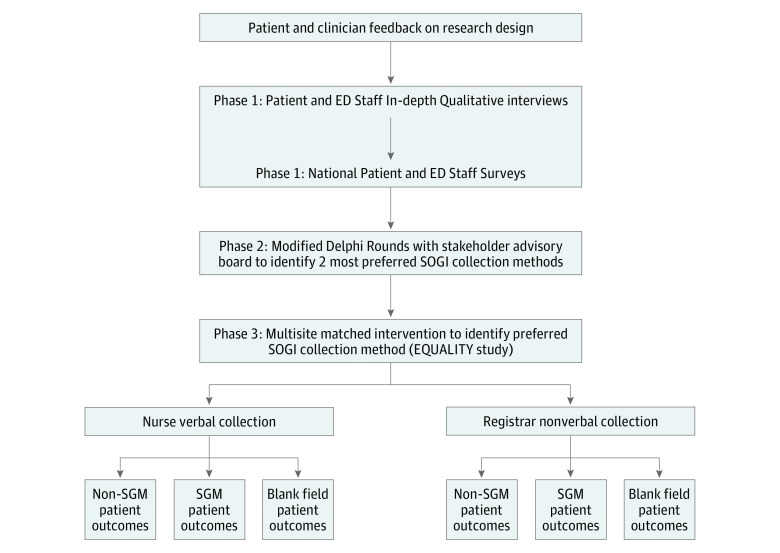
EQUALITY Study Multiphase Design All study activities occurred concurrent with Stakeholder Advisory Board meetings and input. ED indicates emergency department; EQUALITY, Emergency Department Query for Patient-Centered Approaches to Sexual Orientation and Gender Identity; SGM, sexual or gender minority; and SOGI, sexual orientation and gender identity.

The EQUALITY Study used a multiphase mixed-methods design. Phase 1 consisted of qualitative and quantitative data collection designed to identify facilitators, barriers, and preferred methods to collect SOGI in the ED among patients and clinicians; details of phase 1 methods have been published previously.^10^ Phase 2 used data from phase 1 in modified Delphi rounds^12^ in which stakeholder advisory board (SAB) members identified the 2 most preferred methods of SOGI collection: verbal collection by a nurse vs nonverbal collection during patient registration to implement in a study. In phase 3, these methods of SOGI collection were implemented in a multisite, matched intervention study, which is the subject of this article. Figure 1 describes the EQUALITY Study multiphase design and complete details on phase 3. We followed the Strengthening the Reporting of Observational Studies in Epidemiology (STROBE) reporting guideline.

Participation in the study required completion of an informed consent form. The institutional review boards of Partners Healthcare and Johns Hopkins Medicine approved the study protocol. The study was registered at ClinicalTrials.gov (identifier NCT02701049).

### Role of SAB in Design of This Study

A multidisciplinary SAB comprised of patients, physicians, and LGBT health advocates helped to inform our study design. Based on results from phase 1 of the EQUALITY Study, the SAB chose the 2 most viable and preferred methods of SOGI collection, as well as relevant outcome measures to use in the interventional study.

### Intervention

The intervention study was conducted at 2 academic EDs (Johns Hopkins University and Brigham and Women’s Hospital) and 2 community EDs (Howard County and Brigham and Women’s Faulkner). The 2 SOGI collection approaches were nurse verbal collection during the clinical encounter (mode 1) at all sites between February 2016 and March 2017, followed by registrar nonverbal collection of SOGI information during registration (mode 2) between October 2016 and April 2017. The ED physicians, physician assistants, nurses, and registrars received education and training on SGM health disparities and terminology prior to and throughout the intervention period.

During mode 1, ED nurses were requested to collect SOGI information from their patients as part of the social history portion of the patient assessment and enter it directly into the EHR, which had been previously modified to collect this data. During mode 2, registrars asked patients to confidentially complete a demographics information form that included SOGI information, administered via iPad at the 2 sites in Boston, Massachusetts, or on paper at the sites in Baltimore, Maryland. Electronic forms were automatically attached to the patient’s EHR, while paper forms were immediately entered into the EHR by staff. In both modes, researchers ran analytic reports every hour (when on duty from 7 am-10 pm) to identify patients in the ED who were eligible for a survey to ascertain which method of SOGI was preferable to them. Notably, SOGI information collection is currently not a widely accepted standard of care in the ED, so we could not mandate its collection. However, during both modes, nurses and registrars received several education sessions by experts and LGBT patient advocates to explain the need for routine SOGI information collection. We also incentivized staff with gift cards for those who collected this information on the most patients.

### Participants

Adults older than18 years were eligible to have SOGI information collected as part of the intervention. Every patient who identified themselves as a SGM was invited to complete outcome surveys. Sexual and gender minorities patients who consented and were enrolled were matched 1 to 1 by age (aged ≥5 years) and illness severity^13^ (Emergency Severity Index score ±1) to patients that identified as heterosexual and cisgender (non-SGM) and to patients who SOGI information was missing (blank field). Matched non-SGM and blank field patients were also invited to complete outcome surveys. Patients who did not speak English, whose chief concern was psychiatric or alcohol and/or drug-related, or who had an Emergency Severity Index rating of 1 were ineligible.

### Outcomes

The primary outcome was patient satisfaction as measured by a scale modified from the Communication Climate Assessment Toolkit (CCAT) patient survey.^14^ The full CCAT contains 7 items and our modified CCAT contains 5 items that were applicable to the ED population. For example, we kept the question “Do you feel welcome at the hospital?” but eliminated the question “Was it easy to reach someone on the phone if you had a question?” for our analyses. Each scale item had a minimum score of 0 and a maximum score of 1, resulting in a scale score ranging from 0 to 5; higher scores were considered better. The average score for the modified scale was calculated and multiplied by 20 to provide the overall score out of 100. Secondary outcomes assessed patient satisfaction including overall patient comfort, patient experiences, and patient comfort with SOGI collection. Survey items used a Likert-type scale, and responses were grouped into 2 or 3 categories for analysis, eg, very uncomfortable or uncomfortable vs neither comfortable nor uncomfortable, vs comfortable or very comfortable; or not at all concerned vs a little concerned or somewhat concerned or concerned or very concerned. To understand overall acceptability of each method of SOGI collection within staff workflow, we also assessed the proportion of patients from whom SOGI was collected with each collection method.

### Statistical Analysis

To detect a difference of 10 CCAT score points—the smallest change in score that still corresponds to statistically meaningful changes in patients’ beliefs that they are receiving high-quality care^14^—between intervention modes with 90% power, we estimated a priori that we would need to enroll a total of 128 LGBT patients across both SOGI collection approaches. After matching to heterosexual and cisgender patients and blank field patients, the total estimated sample needed to power the study was 384 across both SOGI collection approaches.

Primary and other outcome results were compared between SOGI collection approaches using analysis of variance to test differences between means, or χ^2^ tests to test associations between 2 variables for each patient match group (SGM, non-SGM, and blank field) (both, α < .05). As the modified CCAT is an ordinal categorical variable based on a summation of subset scores, we used multivariable ordered logistic regression to examine whether intervention mode was associated with modified CCAT scores after controlling for potential confounding variables such as race, illness severity (as measured by Emergency Severity Index), and hospital site. Although patients completing outcome surveys were matched on age, we controlled for age as a continuous variable in the regression model to further adjust for any intragroup variation. We also controlled for race using the following 3 categories: white, black, and other.

Additionally, we conducted a number of sensitivity analyses including a standard linear regression model, wait time as a covariate, and an ordered logistic regression using the full CCAT score as the outcome. All statistical analyses were performed using SAS, version 9.4 (SAS Institute) and Stata software (version 14.2; StataCorp LP).

## Results

### Study Participants

**Figure 2.**
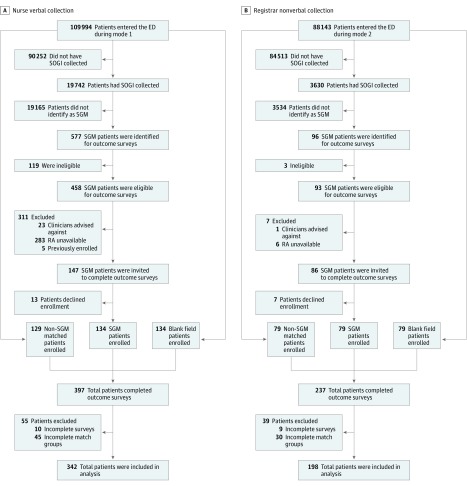
EQUALITY Study Enrollment ED indicates emergency department; EQUALITY, Emergency Department Query for Patient-Centered Approaches to Sexual Orientation and Gender Identity; SGM, sexual or gender minority; and SOGI, sexual orientation and gender identity.

Of the 540 enrolled patients, the mean age was 36.4 years and 66.5% of those who identified their gender were female. Table 1 provides full demographical information on participants. Sexual orientation and gender identity data were collected from 23 372 patients during the intervention period, of whom 673 identified as SGM. Figure 2 provides full details on inclusion, exclusion, and enrollment during each intervention mode. We were able to invite 233 SGM patients to participate in the study, of whom 213 individuals (92%) enrolled and completed outcome surveys. Non-SGM (n = 208) and blank field (n = 213) patients were matched to enrolled SGM patients. Of the 621 patients in fully matched triads, 540 (87%) had complete survey data and were included in final analyses.

**Table 1.** Characteristics of 540 Patients Completing Outcome Surveys, by SGM Patients, Non-SGM Patients, and Blank Field Patients

Patient Characteristics	SGM (n = 180)			Nonsexual and Gender Minorities (n = 180)			Blank Field (n = 180)		
	Mode 1 (n = 114)	Mode 2 (n = 66)	*P* Value^a^	Mode 1 (n = 114)	Mode 2 (n = 66)	*P* Value^b^	Mode 1 (n = 114)	Mode 2 (n = 66)	*P* Value^c^
Age, mean (SD)	38.4 (138)	33.0 (12.9)	.01	38.9 (13.4)	33.1 (12.3)	.004	38.2 (13.4)	33.0 (12.0)	.009
Gender identity, No. (%)^d^									
Male	40 (35.1)	10 (15.1)	.004	30 (26.3)	10 (15.1)	.30	5 (4.3)	1 (1.5)	.99
Female	60 (52.6)	42 (63.6)		80 (70.2)	42 (63.6)		9 (7.9)	4 (6.1)	
Transgender male to female	1 (0.9)	1 (1.5)		0	1 (1.5)		0	0	
Transgender female to male	3 (2.6)	2 (3.0)		0	2 (3.0)		0	0	
Queer/genderqueer	0	3 (4.5)		0	3 (4.5)		0	0	
Questioning/unsure	0	1 (1.5)		0	1 (1.5)		0	0	
Declined to state	0	0		0	0		0	0	
Other	0	0		0	0		0	0	
Missing	10 (8.7)	7 (10.6)		4 (3.5)	7 (10.6)		100 (87.7)	61 (92.4)	
Race, No. (%)									
White	39 (34.2)	30 (45.4)	.07	42 (36.8)	26 (39.4)	.30	42 (36.8)	21 (31.8)	.29
Black	60 (52.6)	23 (34.8)		60 (52.6)	28 (42.4)		58 (50.9)	34 (51.5)	
Asian	1 (0.9)	0		2 (1.7)	1 (1.5)		0	1 (1.5)	
American Indian	1 (0.9)	2 (3.0)		0	1 (1.5)		0	0	
Native Hawaiian	0	1 (1.5)		0	0		0	0	
Hispanic	11 (9.6)	6 (9.1)		8 (7.0)	10 (15.1)		11 (9.6)	9 (13.6)	
Other	2 (1.7)	1 (1.5)		1 (0.9)	0		1 (0.9)	3 (4.5)	
Unknown	0	0		0	0		2 (1.7)	0	
Declined	0	0		1 (0.9)	0		1 (0.9)	1 (1.5)	
Missing	0	0		0	0		0	0	
Emergency Severity Index, No. (%)									
2	14 (12.3)	11 (16.7)	.002	17 (14.9)	8 (12.1)	.001	9 (7.9)	7 (10.6)	.06
3	84 (73.7)	34 (51.5)		87 (76.3)	37 (56.1)		93 (81.6)	44 (66.7)	
4	16 (14.0)	16 (24.2)		9 (7.9)	18 (27.3)		12 (10.5)	15 (22.7)	
5	0	5 (7.6)		1 (0.9)	3 (4.5)		0	0	
Missing	0	0		0	0		0	0	
Study site, No. (%)									
Site 1	28 (24.6)	17 (25.8)	.02	28 (24.6)	17 (25.8)	.02	28 (24.6)	17 (25.8)	.02
Site 2	16 (14.0)	8 (12.1)		16 (14.0)	8 (12.1)		16 (14.0)	8 (12.1)	
Site 3	65 (57.0)	29 (43.9)		65 (57.0)	29 (43.9)		65 (57.0)	29 (43.9)	
Site 4	5 (4.4)	12 (18.2)		5 (4.4)	12 (18.2)		5 (4.4)	12 (18.2)	

Abbreviation: SGM, sexual and gender minorities.

^a^ *P* values represent the difference between modes 1 and 2 among SGM patients who enrolled in the study.

^b^ *P* values represent the difference between modes 1 and 2 among non-SGM patients who enrolled in the study.

^c^ *P* values represent the difference between modes 1 and 2 among blank field patients who enrolled in the study.

^d^ Gender identity as reported in 2-step method (1, assigned sex at birth; 2, current gender identity).

### Patient Outcomes

Mean modified CCAT scores were 6 points higher among SGM patients whose SOGI information was collected by form during registration compared with nurse verbal collection (mean [SD], 95.6 [11.9] vs 89.5 [20.5]; *P* = .03) (Table 2). No significant differences between the 2 approaches were found among non-SGM patients (mean [SD], 91.8 [18.9] vs 93.2 [13.6]; *P* = .59) or those with a blank field (92.7 [15.9] vs 93.6 [14.7]; *P* = .70). No significant differences were found among SGM patients between modes 1 and 2 on the secondary outcomes (Table 3).

**Table 2.** Primary Outcomes Among SGM Patients, Non-SGM Patients, and Blank Field Patients (N = 540)

Study Outcome	SGM Patients (n = 180)			Non-SGM Patients (n = 180)			Blank Field Patients (n = 180)		
	Mode 1 (n = 114)	Mode 2 (n = 66)	*P* Value^a^	Mode 1 (n = 114)	Mode 2 (n = 66)	*P* Value^b^	Mode 1 (n = 114)	Mode 2 (n = 66)	*P* Value^c^
Modified CCAT score (5 items), mean (SD)^d^	89.5 (20.5)	95.6 (11.9)	.03	91.8 (18.9)	93.2 (13.6)	.59	92.7 (15.9)	93.6 (14.7)	.70
CCAT score (5 items), No. (%) of patients^e^						.27^f^			.63^f^
0	1 (0.9)	0	.55^f^	1 (0.9)	0		0	0	
10	0	0		0	0		1 (0.9)	0	
20	1 (0.9)	1 (1.5)		2 (1.7)	0		0	0	
30	3 (2.6)	0		1 (0.9)	0		1 (0.9)	0	
40	3 (2.6)	0		3 (2.6)	1 (1.5)		0	0	
50	2 (1.7)	0		0	2 (3.0)		4 (3.5)	4 (6.1)	
60	4 (3.5)	0		1 (0.9)	1 (1.5)		3 (2.6)	3 (4.5)	
70	2 (1.7)	2 (3.0)		3 (2.6)	3 (4.5)		4 (3.5)	1 (1.5)	
80	9 (7.9)	4 (6.1)		11 (9.6)	3 (4.5)		5 (4.4)	2 (3.0)	
90	13 (11.4)	7 (10.6)		8 (7.0)	10 (15.1)		13 (11.4)	3 (4.5)	
100	76 (66.7)	52 (78.8)		84 (73.7)	46 (69.7)		83 (72.8)	53 (80.3)	

Abbreviations: CCAT, Communication Climate Assessment Toolkit; SGM, sexual and gender minorities.

^a^ *P* values represent the difference between modes 1 and 2 among SGM patients who enrolled in the study.

^b^ *P* values represent the difference between modes 1 and 2 among non-SGM patients who enrolled in the study.

^c^ *P* values represent the difference between modes 1 and 2 among blank field patients who enrolled in the study.

^d^ The full CCAT patient survey contains 7 items and our modified CCAT contains 5 items that are applicable to the emergency department population. Each scale item had a minimum score of 0 and a maximum score of 1, resulting in a scale score ranging from 0 to 5; higher scores were considered better. The mean score for the modified scale was calculated and multiplied by 20 to provide the overall score out of 100.

^e^ The CCAT (5 items) is an ordinal categorical variable; this shows where each patient fell in each of the 11 possible score categories.

^f^ Fisher exact test results reported.

**Table 3.** Secondary Outcomes Among SGM Patients, Non-SGM Patients, and Blank Field Patients (N = 540)

Study Outcome	SGM Patients (n = 180)			Non-SGM Patients (n = 180)			Blank Field Patients (n = 180)		
	Mode 1 (n = 114)	Mode 2 (n = 66)	*P* Value^a^	Mode 1 (n = 114)	Mode 2 (n = 66)	*P* Value^b^	Mode 1 (n = 114)	Mode 2 (n = 66)	*P* Value^c^
Staff comfort with patient, No. (%)									
Uncomfortable or very uncomfortable	21 (18.3)	12 (18.2)	.50^d^	23 (20)	15 (22.7)	.64^d^	21 (18.3)	6 (9.1)	.22^d^
Neither comfortable nor uncomfortable	7 (6.1)	1 (1.5)		5 (4.3)	1 (1.5)		4 (3.5)	1 (1.5)	
Comfortable or very comfortable	85 (74.6)	53 (80.3)		86 (75.4)	50 (75.8)		89 (78.1)	58 (87.9)	
Did not answer	1 (0.9)	0		0	0		0	1 (1.5)	
Staff treat patient with respect, No. (%)									
Disrespectful or very disrespectful	12 (10.4)	4 (6.1)	.57^d^	11 (9.6)	4 (6.1)	.31^d^	9 (7.8)	2 (3.0)	.07^d^
Neutral	7 (6.1)	3 (4.5)		8 (7.0)	2 (3.0)		9 (7.8)	1 (1.5)	
Respectful or very respectful	94 (82.5)	59 (89.4)		95 (83.3)	59 (89.4)		96 (84.2)	63 (95.4)	
Did not answer	1 (0.9)	0		0	1 (1.5)		0	0	
Staff ignore patient, No. (%)									
Never/rarely	101 (87.8)	61 (92.4)	.92^d^	97 (85.1)	61 (92.4)	.19^d^	101 (88.6)	63 (95.4)	.45^d^
Sometimes	8 (7.0)	3 (4.5)		12 (10.5)	3 (4.5)		9 (7.9)	3 (4.5)	
Often/constantly	3 (2.6)	1 (1.5)		5 (4.4)	1 (1.5)		2 (1.7)	0	
Did not answer	2 (1.7)	1 (1.5)		0	1 (1.5)		2 (1.7)	0	
Patient concerned about privacy, No. (%)									
Not at all concerned	84 (73.7)	53 (80.3)	.54^d^	94 (82.5)	51 (77.3)	.56^d^	94 (82.5)	52 (78.8)	.40^d^
A little/somewhat/very concerned	27 (23.7)	11 (16.7)		20 (17.5)	14 (21.2)		19 (16.7)	13 (19.7)	
Did not answer	3 (2.6)	2 (3.0)		0	0		1 (0.9)	1 (1.5)	
Piece of personal information least comfortable sharing, No. (%)									
Race/ethnicity	2 (1.7)	4 (6.1)	.44^d^	4 (3.5)	1 (1.5)	.32^d^	0	0	.45^d^
Income	6 (5.3)	2 (3.0)		3 (2.6)	5 (7.6)		2 (1.7)	0	
Sexual orientation	16 (14.0)	10 (15.1)		5 (4.4)	4 (6.1)		4 (3.5)	0	
Gender Identity	9 (7.9)	3 (4.5)		5 (4.4)	2 (3.0)		1 (0.9)	0	
Religion	5 (4.4)	2 (3.0)		5 (4.4)	5 (7.6)		2 (1.7)	2 (3.0)	
Other	11 (9.6)	12 (18.2)		13 (11.4)	12 (18.2)		9 (7.9)	3 (4.5)	
Not applicable	60 (52.6)	32 (48.5)		75 (65.8)	34 (51.5)		94 (82.5)	60 (90.9)	
Did not answer	5 (4.4)	1 (1.5)		4 (3.5)	3 (4.5)		2 (1.7)	1 (1.5)	
Comfort reporting sexual orientation, No. (%)									
Not at all comfortable	4 (3.5)	4 (6.1)	.05^d^	2 (1.7)	3 (4.5)	<.001^d^	0	0	.57^d^
A little/somewhat comfortable	13 (11.4)	14 (21.2)		5 (4.4)	4 (6.1)		3 (2.6)	1 (1.5)	
Comfortable or very comfortable	77 (67.5)	44 (66.7)		56 (49.1)	49 (74.2)		30 (26.3)	22 (33.3)	
Did not answer	20 (17.5)	4 (6.0)		51 (44.7)	10 (15.1)		81 (71.1)	43 (65.2)	
Comfort reporting gender identity, No. (%)									
Not at all comfortable	2 (1.7)	2 (3.0)	.05^d^	2 (1.7)	2 (3.0)	<.001^d^	1 (0.9)	0	.71^d^
A little/somewhat comfortable	11 (9.6)	4 (6.1)		4 (3.5)	2 (3.0)		1 (0.9)	1 (1.5)	
Comfortable or very comfortable	77 (67.5)	55 (83.3)		61 (53.5)	54 (81.8)		36 (31.6)	25 (37.9)	
Did not answer	24 (20.9)	5 (7.6)		47 (40.9)	8 (12.1)		76 (66.7)	40 (60.6)	
Important for all patients to provide sexual orientation, No. (%)									
Not at all important	30 (26.1)	18 (27.3)	.12	37 (32.5)	19 (28.8)	.04	46 (40.3)	21 (31.8)	.03
A little/somewhat important	23 (20.2)	14 (21.2)		17 (14.9)	9 (13.6)		17 (14.9)	15 (22.7)	
Important or very important	47 (41.2)	18 (27.3)		50 (43.9)	22 (33.3)		38 (33.3)	14 (21.2)	
Did not answer	14 (12.2)	16 (24.2)		10 (8.7)	16 (24.2)		13 (11.3)	16 (24.2)	
Important for all patients to provide gender identity, No. (%)									
Not at all important	25 (21.9)	12 (18.2)	.23	26 (22.8)	12 (18.2)	.16	43 (37.7)	15 (22.7)	.02
A little/somewhat important	23 (20.2)	11 (16.7)		21 (18.4)	11 (16.7)		20 (17.5)	17 (25.8)	
Important or very important	52 (45.6)	27 (40.9)		54 (47.4)	27 (40.9)		39 (34.2)	18 (27.3)	
Did not answer	14 (12.2)	16 (24.2)		13 (11.3)	16 (24.2)		12 (10.4)	16 (24.2)	

Abbreviation: SGM, sexual and gender minorities.

^a^ *P* values represent the difference between modes 1 and 2 among SGM patients who enrolled in the study.

^b^ *P* values represent the difference between modes 1 and 2 among non-SGM patients who enrolled in the study.

^c^ *P* values represent the difference between modes 1 and 2 among blank field patients who enrolled in the study.

^d^ Fisher exact test results reported.

Because we were interested in the experiences of SGM patients, we conducted an independent ordered logistic regression analysis for each of the patient match groups (SGM, non-SGM, and blank field). In unadjusted regression models stratifying by patient match group, SGM patients had 1.98 times the odds of having a higher CCAT score between modes 1 and 2 (95% CI, 0.99-3.98). After adjusting for age, race, illness severity, and study site the strength of the association increased to 2.57 (95% CI, 1.13-5.82) (Table 4). The odds of a better CCAT score between modes 1 and 2 among non-SGM or blank field patients were not significant (Table 4). Note, because these analyses were performed within, but not across, each of the 3 groups, adjusted analyses controlled for factors on which the groups had been matched, including age and injury severity.

**Table 4.** Odds of Increasing Modified CCAT Scores by Intervention Mode, Patient, and Hospital Characteristics

Patient and Hospital Characteristics	OR (95% CI)^a^	
	Unadjusted	Adjusted^b^
SGM patients (n = 180)		
Data collection method [reference = nurse verbal]	1.98 (0.99-3.98)	2.57 (1.13-5.82)
Age	0.99 (0.97-1.02)	0.99 (0.97-1.02)
Race [reference = white]		
Black	0.88 (0.44-1.74)	1.15 (0.51-2.58)
Other	1.37 (0.48-3.88)	1.62 (0.53-4.96)
Emergency Severity Index [reference = 2]		
3	0.82 (0.30-2.22)	1.03 (0.35-3.00)
4	0.78 (0.24-2.50)	0.70 (0.20-2.43)
5	0.46 (0.06-3.39)	0.25 (0.03-2.27)
Site [reference = 1]		
2	1.35 (0.42-4.38)	1.58 (0.46-5.39)
3	0.80 (0.36-1.73)	0.86 (0.33-2.21)
4	0.77 (0.24-2.45)	0.49 (0.13-1.84)
Non-SGM patients (n = 180)		
Data collection method [reference = nurse verbal]	0.90 (0.47-1.73)	0.87 (0.42-1.83)
Age	1.02 (0.99-1.05)	1.02 (0.99-1.05)
Race [reference = white]		
Black	1.10 (0.55-2.17)	1.65 (0.78-3.50)
Other	2.18 (0.66-7.13)	2.82 (0.79-10.06)
Emergency Severity Index [reference = 2]		
3	0.44 (0.14-1.36)	0.59 (0.17-1.98)
4	0.48 (0.12-1.81)	0.63 (0.14-2.83)
5	0.46 (0.04-5.70)	0.67 (0.05-9.70)
Site [reference = 1]		
2	0.67 (0.20-2.17)	0.75 (0.22-2.63)
3	0.37 (0.16-0.89)	0.40 (0.16-1.04)
4	0.84 (0.20-3.65)	1.08 (0.24-4.92)
Blank field patients (n = 180)		
Data collection method [reference = nurse verbal]	1.41 (0.68-2.93)	1.72 (0.78-3.79)
Age	1.01 (0.99-1.04)	1.01 (0.98-1.04)
Race [reference = white]		
Black	0.58 (0.27-1.24)	0.68 (0.29-1.59)
Other	0.96 (0.30-3.05)	0.76 (0.22-2.61)
Emergency Severity Index [reference = 2]		
3	1.50 (0.49-4.55)	1.42 (0.43-4.74)
4	1.25 (0.34-4.61)	1.15 (0.28-4.61)
5	NA^c^	NA^c^
Site [reference = 1]		
2	1.84 (0.46-7.41)	1.79 (0.42-7.63)
3	0.70 (0.31-1.59)	0.81 (0.32-2.06)
4	0.62 (0.18-2.15)	0.52 (0.14-2.00)

Abbreviations: CCAT, Communication Climate Assessment Toolkit; NA, not applicable; OR, odds ratio; SGM, sexual and gender minorities.

^a^ Odds of changing to one higher category of modified CCAT score

bBecause these analyses were performed within, but not across, each of the 3 groups, adjusted analyses controlled for factors on which the groups had been matched, including age and injury severity.

^c^ No blank field patients with Emergency Severity Index (rating of 5).

We also found that most patients said it was important for all patients to report SOGI information: 62.4% of patients reported that it was important for all patients to provide sexual orientation information (95% CI, 0.580-0.669; *P* < .001) and 70.6% of patients reported that it was important for all patients to provide gender identity information (95% CI, 0.664-0.748; *P* < .001). Further analyses demonstrated no significant differences in comfort reporting SOGI across patient modes and among SGM, non-SGM, and blank field patients.

### Sensitivity Analyses

We examined ED wait time as a potential indicator of CCAT scores as wait time has been shown to be a potential indicator of satisfaction with ED encounters.^15,16^ However, because our analysis showed wait time was not a significant potential indicator of modified CCAT score overall or in the stratified analyses, we excluded it from the final model. In addition, similar findings were observed when we repeated each of our ordered logistic regression analyses, which had examined the modified CCAT score as ordered categorical variable, using standard linear regression models where the modified CCAT score was included as a continuous variable.

## Discussion

Our interventional study assessing 2 potential methods to collect SOGI in the ED found that SGM patients reported significantly higher satisfaction with their experience in the ED with registrar nonverbal collection compared with nurse verbal collection. In other words, SGM patients preferred a standardized collection process where all patients could report SOGI along with other demographical information vs being asked by a nurse during a clinical encounter. Non-SGM patients and those without reported SOGI information were no less satisfied with form collection compared with verbal collection.

By using a mixed-methods approach, the EQUALITY Study aimed to assess whether a truly patient-centered method to collect SOGI. Early qualitative work from the study revealed a dichotomy between patients and physicians and nurses: while only 10% of patients reported they would refuse to provide sexual orientation in the ED, nearly 80% of clinicians expected that patients would refuse to answer such a question.^10^ Patients are willing to provide SOGI information, especially if collected in a standardized manner emphasizing population health.^11^ These findings were consistent with opinions voiced during our SAB meetings. Members of the SAB and our patient coinvestigators provided critical feedback for designing a successful study. By engaging the SAB via a Delphi approach to identify intervention modes for the study, we hoped to compare modes that were relevant and patient centered. The SAB also guided the development and implementation of staff training modules that were implemented prior to each intervention mode. We believe that these steps resulted in a patient-centered study that effectively evaluated whether patient satisfaction differed between SOGI collection methods.

Many have called for routine SOGI collection to create health care environments that facilitate disclosure and recognition of SGM patients,^17,18,19^ and Meaningful Use Stage 3 Guidelines set the expectation that health care systems should have the ability to collect these data. In addition, previous studies found that most patients believe SOGI collection in the primary clinic setting is important and are willing to provide the information.^20^ Our work extends these findings to the ED, the source of nearly half of inpatient admissions in the United States and a major source of care for uninsured and underinsured patients, many of whom are SGM.^21,22^ Even still, few hospital EDs routinely collect SOGI information.^23^ Most patients report comfort with their ED experience when asked to provide SOGI information in the ED setting.^10^

Our finding that patient satisfaction differed only among SGM patients in a racially diverse sample points to the strength of our results; that is, patients most affected by SOGI collection reported higher satisfaction with the health care encounter during nonverbal self-report. Further, no other outcomes showed nonverbal self-report to be inferior to nurse verbal SOGI collection. Specifically, most patients felt that staff were comfortable interacting with them and staff treated them with respect. Additionally, the majority were not at all concerned about their privacy and felt comfortable reporting SOGI. These findings support nonverbal self-report as an acceptable and feasible method of SOGI collection among both SGM and non-SGM patients.

Although SOGI collection from all patients was lower during nonverbal self-report, this can be explained by the nature of the interventions. Nurse verbal collection was integrated into the EHR and workflow at every study site, and was promoted as a new standard but not yet mandatory practice by the hospital systems. In contrast, registrar nonverbal collection was a new process at every site, requiring workflow reorganization and modification in addition to staff training on SGM health. This finding speaks to the importance of integrating SOGI collection smoothly into workflows and EHRs.

### Limitations

This study had limitations. First, collection of SOGI information in the ED was not compulsory, which resulted in low SOGI collection rates for both verbal and nonverbal methods. Given that we achieved a priori sample sizes, it is unlikely that this affected our major finding as we found no systematic biases in data collection other than staff inclination to collect data. During a poststudy debriefing, nurses and registrars stated that as SOGI collection was compulsory, it was easy to skip to save time in the busy ED environment. This is not surprising in the context of work by Callahan et al^24^ describing the challenges associated with collecting SOGI information in clinical settings. These low overall SOGI collection rates reinforce the need to develop guidelines and patient-centered methods to collect these important data before it becomes a national requirement. Second, we were limited by the answer options that each hospital system chose for its SOGI questions; therefore, we could have missed patients who do not choose to define themselves in a particular SGM category. Additionally, any patient with a psychiatric diagnosis was not eligible for inclusion in the study. Because SGM patients have increased risk of poor mental health,^5^ we may have missed SGM patients who may benefit most from increased SGM sensitivity. Generalizability of these findings to settings outside the ED is difficult to discern, as patients might experience a higher acuity of injury and less time to interface with hospital staff as compared with ambulatory settings. To our knowledge, this is the first study of its kind to evaluate SOGI collection in an acute care setting, and further studies are required in ambulatory settings to allow more staff to patient interaction time and look for similar results. Although geographical generalizability may be a concern, this study took place in both academic and community hospitals in the northeastern and mid-atlantic United States, which confirmed the qualitative themes on the national level.

## Conclusions

Previous research has indicated that many SGM patients feel that disclosing SOGI to a clinician is as difficult as disclosing to other people in their lives,^25^ so the findings of our study are critical to implementing SOGI collection in a patient-centered manner. Results also highlight the necessity for patients to be able to opt out of reporting SOGI information, and for nurses and registrars to receive cultural sensitivity and dexterity training on caring for patients who disclose that they identify as SGM.^26^ Sexual and gender minorities patients deserve to be recognized, be acknowledged, and feel comfortable in the health care setting. The EQUALITY Study shows that nonverbal self-report of SOGI is the clear patient-centered method associated with higher SGM patient satisfaction in the ED. Non-SGM patients are equally comfortable with this approach. Based on these data, we recommend adoption of nonverbal self-report to collect SOGI information in ED settings.
